# Differential Utilization of Dietary Fatty Acids in Benign and Malignant Cells of the Prostate

**DOI:** 10.1371/journal.pone.0135704

**Published:** 2015-08-18

**Authors:** Andrea Dueregger, Bernd Schöpf, Theresa Eder, Julia Höfer, Erich Gnaiger, Astrid Aufinger, Lukas Kenner, Bernhard Perktold, Reinhold Ramoner, Helmut Klocker, Iris E. Eder

**Affiliations:** 1 Division of Experimental Urology, Department of Urology, Medical University of Innsbruck, Innsbruck, Austria; 2 Oncotyrol GmbH, Center for Personalized Medicine, Innsbruck, Austria; 3 Division of Genetic Epidemiology, Department of Medical Genetics, Molecular and Clinical Pharmacology, Medical University of Innsbruck, Innsbruck, Austria; 4 Oroboros Instruments, High-Resolution Respirometry, Innsbruck, Austria; 5 Clinical Institute for Pathology, Medical University Vienna, Vienna, Austria; 6 Diätologie, FHG-Zentrum Für Gesundheitsberufe Tirol GmbH, Innsbruck, Austria; 7 Department of General and Transplant Surgery, D. Swarovski Research Laboratory, Medical University of Innsbruck, Innrain 66/6, A-6020, Innsbruck, Austria; University of Kentucky College of Medicine, UNITED STATES

## Abstract

Tumor cells adapt via metabolic reprogramming to meet elevated energy demands due to continuous proliferation, for example by switching to alternative energy sources. Nutrients such as glucose, fatty acids, ketone bodies and amino acids may be utilized as preferred substrates to fulfill increased energy requirements. In this study we investigated the metabolic characteristics of benign and cancer cells of the prostate with respect to their utilization of medium chain (MCTs) and long chain triglycerides (LCTs) under standard and glucose-starved culture conditions by assessing cell viability, glycolytic activity, mitochondrial respiration, the expression of genes encoding key metabolic enzymes as well as mitochondrial mass and mtDNA content. We report that BE prostate cells (RWPE-1) have a higher competence to utilize fatty acids as energy source than PCa cells (LNCaP, ABL, PC3) as shown not only by increased cell viability upon fatty acid supplementation but also by an increased ß-oxidation of fatty acids, although the base-line respiration was 2-fold higher in prostate cancer cells. Moreover, BE RWPE-1 cells were found to compensate for glucose starvation in the presence of fatty acids. Of notice, these findings were confirmed *in vivo* by showing that PCa tissue has a lower capacity in oxidizing fatty acids than benign prostate. Collectively, these metabolic differences between benign and prostate cancer cells and especially their differential utilization of fatty acids could be exploited to establish novel diagnostic and therapeutic strategies.

## Introduction

Prostate cancer (PCa) is among the most commonly diagnosed cancers in Western countries [1,2]. Its strong dependence on hormones renders endocrine therapy the most important treatment modality, especially in patients with more advanced stages of the disease (reviewed in [3]). Despite good initial efficacy, however, androgen deprivation therapy is merely palliative since most patients eventually experience castration-resistant PCa (CRPC) (reviewed in [4,5]). A substantial proportion of patients ultimately relapse with metastatic disease, which is typically associated with poor prognosis and limited therapeutic options (reviewed in [6]).

Due to continuous proliferation, tumor cells are challenged to meet their increased energy requirements (reviewed in [7]), a phenomenon first described in the early 1920s by Otto Warburg [8]. Most healthy cells fulfill their energy needs via oxidative phosphorylation (OXPHOS) whereby glucose is metabolized to pyruvate, which is further oxidized through the tricarboxylic acid cycle (TCA) in the mitochondria, yielding ~ 34 ATPs. The “Warburg effect” states that upon malignant transformation, cells switch to aerobic glycolysis, identified by an increased glucose consumption and lactate production, also under sufficient oxygen supply. This fast generation of two ATPs via glycolysis was originally thought to compensate for an ATP loss by defective mitochondrial OXPHOS. However, Warburg´s initial hypothesis has recently been revised by findings that cancer cells do not necessarily exhibit impaired mitochondrial function and that mitochondrial OXPHOS persists in most tumors instead (reviewed in [9]). Thus, data now support the concept of “metabolic reprogramming” in tumor cells where increased aerobic glycolysis is not used instead of but in addition to OXPHOS providing high yields of energy. Indeed, it is known that many types of cancers including breast cancer have increased glycolytic activity compared to their tissue of origin (reviewed in [10]).

PCa cells, on the other hand, were shown to preferentially use fatty acids (FAs) over glucose to fulfill their energy demands [11]. Indeed, altered lipid metabolism has been increasingly recognized as a hallmark of cancer. *De novo* synthesis of FAs is required for membrane synthesis and therefore for cell growth and proliferation. FA synthesis by fatty acid synthase (FASN) is an anabolic process that is increased in many types of cancers, including that of the prostate (reviewed in [12]). Increased activity of lipogenic enzymes was associated with PCa carcinogenesis as well as with metastasis, worse prognosis and poor survival (reviewed in [13]).

The knowledge about metabolic changes in cancer cells has ultimately led to the establishment of various therapeutic applications, including inhibition of glycolysis with specific inhibitors and ketogenic diets (reviewed in [14]). The latter aims to restrict the supply of glucose whilst supplementing high amounts of FAs to furnish the body with adequate energy. A significant number of studies and reviews have provided evidence that dietary FAs may play a role in the etiology of PCa [15–17]. Most research in this field focused on investigating the effects of long chain triglycerides (LCTs) showing that omega-3 LCTs, for instance, exert a protective effect on cancer risk [18,19]. In addition, ketogenic diets rich in omega-3 LCTs and medium chain triglycerides (MCTs) showed inhibitory effects on various cancers [20–22]. LCTs and MCTs are catabolized via the β-oxidation pathway in the mitochondria after entering the cell either via FA protein transporters or via direct diffusion, respectively. Inside the cell, FAs undergo different metabolic fates depending on their chain length. LCTs require carnitine palmitoyltransferase 1 (CPT1) conversion of the long chain acyl-CoA to long chain acyl carnitine to be transported into the mitochondria. By contrast, MCTs do not require this shuttle system to penetrate mitochondria [23]. Through the mitochondrial β-oxidation pathway, FAs are oxidized to acetyl CoA, a process resulting in a net production of twice as much ATP compared to glucose catabolism. Moreover, upon starvation, ketone bodies may be produced from FAs as a compensatory cellular energy source [24].

This study aimed to investigate differences in the bioenergetics utilization of glucose and dietary FAs between BE and cancer cells of the prostate. In particular, we studied the effects of MCTs and LCTs on cell viability under normal and reduced glucose conditions, glycolytic activity, mitochondrial OXPHOS, as well as mitochondrial number and mtDNA content. In addition, we performed OXPHOS measurements in human tissue samples, which confirm our in vitro findings. In brief, our data revealed that BE prostate epithelial cells have a higher affinity to utilize dietary FAs than PCa cells under standard culture condition as well as under glucose starvation.

## Materials and Methods

### Reagents and cell lines

Reagents were purchased from Sigma Aldrich (St. Louis, MO) unless otherwise specified. MCT, ω3-LCT and combinations thereof (summarized in Table 1) were provided by Dr. Schär AG/SPA (Burgstall, Italy). All oils were brought to a stock solution of 40 mM by diluting them in DMSO. PCa (LNCaP and PC3), benign prostate (RWPE-1) cell lines were purchased from the American Type Culture Collection (ATCC; Rockville, MD). The castration resistant PCa subline LNCaPabl (ABL) was established from LNCaP cells by *in vitro* long-term androgen ablation as described previously [25]. All cell lines were maintained in DMEM medium with a glucose level of 1g/L and 10% fetal calf serum (Gibco BRL, Life technologies) at 37°C in a humidified atmosphere with 5% CO_2_. Cell lines and their growth characteristics are listed in Table 2. DuCaP were obtained from Prof. J. Schalken (Center for Molecular Life Science, Nijmegen, The Netherlands) and were maintained as previously described [26].

**Table 1:** Composition of different oils employed in this study.

Oil	Fatty acid composition (%)	Components	FA
MCT/LCT	77%/23%	coconut oil, palm oil, thistle oil, line seed oil	lauric acid (C-10), linoleic acid, alpha-linoleic acid (C-22)
MCT	100%	coconut oil, palm oil	lauric acid (C-10)
LCT	100%	thistle oil, line seed oil	linoleic acid, alpha-linoleic acid (C-22)

DMSO was used as solvent to add the different oils to culture the media (20 mM stock solutions).

**Table 2:** Cell lines used in this study.

Histological origin	Status	Cell line	Hormone dependence
prostate	benign	RWPE-1	responsive
	carcinoma	PC3	independent
	carcinoma	LNCaP	dependent
	carcinoma	ABL^a^	independent
	carcinoma	DuCaP	dependent

^a^ ABL are a sub-cell line of LNCaP that were grown for 60 passages without androgen hormones generating a model cell line for castration resistant prostate CA (CRPC) that is hormone independent for growth [25]

### Cell viability assay

Cells were seeded into 96 well plates over night before addition of the substances at indicated concentrations. Cell viability was assessed after 72 h by WST-1 assay (Roche, Basel, Switzerland) as described in detail previously [27].

### Measurement of glucose uptake and lactate production

The amount of glucose was measured with a colorimetric assay as originally described [28]. In brief, the measurement is based on the conversion of glucose to glucose 6-phophate, which is then converted to 6-phosphogluconate generating NADPH. NADPH reduces N-methylphenazonium methyl sulphate (PMS) to PMSH, which in turn reduces piodonitrotetrazolium violet INT to INTH. Absorbance is measured at 450–520 nm. Lactate was determined using a colorimetric assay as described by Babson [29]. This assay is based on the conversion of lactate to pyruvate by lactate dehydrogenase (LDH) thereby reducing NAD to NADH, consequently resulting in the reduction of the colorimetric dye INT to INTH, which is then measured at 450–520 nm. Glucose uptake and lactate production were calculated following subtraction of background values and data normalized to cell number.

### High-resolution respirometry (HRR)

Respirometric experiments were performed at 37°C in a glass chamber of an OROBOROS Oxygraph under normoxic (200 μM O_2_ for cells) or high-oxygen (300–200 μM O_2_ for tissue) conditions. DatLab software (OROBOROS INSTRUMENTS, Innsbruck, Austria) was used for real-time data acquisition and analysis. The titration protocol used for the experiments has been reported previously and was used with some minor modifications [30].

In brief, cells were trypsinized, washed with PBS and resuspended in mitochondrial respiration medium (MiR05: 110 mM sucrose, 60 mM K ^+^-lactobionate, 0.5 mM EGTA, 3 mM MgCl_2_, 20 mM taurine, 10 mM KH_2_PO_4_, 20 mM HEPES adjusted to pH 7.1 with KOH at 37°C; and 1 g/L BSA essentially fatty acid free [31]) to obtain a concentration of 0.5x10^6^ cells per mL. After permeabilization with digitonin (10 μg per 10^6^ cells), octanoyl-carnitine (5 mM) and malate (0.4 mM) were added and fatty acid ß-oxidation (FAO) linked oxidative phosphorylation was measured after the titration of ADP (1 mM for cells, 2.5 mM for tissue, respectively).

Subsequently, glutamate (10 mM) and pyruvate (5 mM) were added as a source of NADH and thus linked to Complex I (CI). Cytochrome *c* (10 μM) was applied to control for outer mitochondrial membrane integrity. The cytochrome *c* test was followed by the addition of succinate (10 mM) to assess CI&II&FAO-linked respiration. Uncoupling was performed by stepwise titration of carbonyl cyanide m-chloro phenyl hydrazine (CCCP, 0.5 μM steps) and followed by CI inhibition (rotenone, 0.5 μM). Finally, CII and CIII were inhibited by addition of malonate (5 mM) succeeded by antimycin A (2.5 μM) to correct for residual oxygen consumption. Respiratory flow, *I*_O2_, is expressed as pmol O_2_·s^-1^·10^−6^ cells.

Tissue specimens were harvested directly after radical prostatectomy by extracting one biopsy predominantly consisting of cancer cells and one control biopsy containing only BE tissue for each subject to be analyzed (Table 3, S1 Fig). The use of human prostate tissue for high-resolution respirometry was approved by the ethical committee of the Medical University of Innsbruck (protocol no. AN 4837). Written informed consent was obtained from all patients (n = 6). A small portion of the biopsies was frozen for subsequent hematoxylin/eosin (HE) staining and p63/P504S immunohistochemistry to confirm the presence or absence of tumor cells. The remaining tissue sections were placed immediately into ice-cold relaxing and preservation solution BIOPS, containing 2.77 mM CaK_2_EGTA, 7.23 mM K_2_EGTA, 20 mM imidazole, 20 mM taurine, 50 mM MES hydrate, 0.5 mM DTT, 6.56 mM MgCl_2_, 5.77 mM ATP and 15 mM phosphocreatine for transportation [31,32]. For permeabilization, tissue samples were placed into pre-chilled respiration medium MiR05Cr (MiR05 + creatine) on ice. Mechanical permeabilization was performed in a small glass petri dish on a pre-cooled metal plate using two pairs of very sharp forceps as described previously [33]. After permeabilization, the tissue samples were rinsed once with ice-cold MiR05Cr, blotted for 5 s on a filter paper before wet weight (*W*_*W*_) was determined on a Mettler Toledo XS105DU microbalance. Tissue slices with a *W*_*W*_ ranging from 5–10 mg were used for one single experiment and oxygen consumption was expressed as oxygen flux per tissue mass (*J*_*O2*_, pmol O_2_·s^-1^·mg^-1^).

**Table 3:** Subject characteristics.

	Mean, median (range)
Age at diagnosis [years]	60.0, 62.5 (51.3–67.9)
PSA [ng/ml]	8.3, 7.0 (2.4–18.8)
Prostate weight [g]	36.0, 36.5 (26.0–45.0)
Diagnostic Gleason score (Type I + II)	3 + 4
ERG expression (IHC) positive	4
ERG expression (IHC) negative	2

### Real-time quantitative PCR (qPCR)

RNA isolation, cDNA synthesis and qPCR were performed as described previously [34]. TaqMan gene expression assays for quantification of pyruvate dehydrogenase kinase 1 (PDK1, Hs01561850_m1), carnitine palmitoyltransferase 1 (CPT1, Hs00157079_m1) and ketone body metabolizing enzymes (3-hydroxy-butyrate dehydrogenase—type 1 and 2: BDH1, Hs00983007_m1; BDH2, Hs01010096_g1; acetoacetyl-CoA synthetase: AACS, Hs00225090) were from Applied Biosystems, Vienna, Austria. Fold change in gene expression was determined using the mathematical model ratio 2^-ΔΔCT^ [35]. Values were normalized to the housekeeping gene HMBS (hydroxymethyl-bilane synthase).

### Determination of mtDNA

Total genomic DNA was extracted on an EZ1 Advanced workstation using the EZ1 DNA Tissue Kit (QIAGEN, Vienna, Austria) according to the manufacturer’s protocol and quantified spectrophotometrically with an Infinite 200 PRO NanoQuant system (Tecan Group Ltd, Maennedorf, Switzerland). Mitochondrial DNA (mtDNA) copy number was measured by qPCR on a 7900HT Fast Real-Time PCR System (Applied Biosystems, Vienna, Austria) using the primer sequences reported [36] (Table 4). For each sample, two mtDNA (COX1 and ND3) and two nuclear DNA (POLG and RRM2B) fragments were amplified and quantified separately. All reactions were carried out in a final volume of 10 μl containing 1ng of DNA, 1 x PerfeCTa SYBRGreen SuperMix, ROX (VWR International, Vienna, Austria) and 150 nM of each primer. Cycling conditions were 95°C for 2 min followed by 40 cycles of 95°C for 15 s, 63°C for 40 s and 72°C for 10 s. Each fragment was amplified in four replicates. For the determination of the mtDNA copy number, ΔCT values (ct_mtDNA_−ct_nDNA_) were calculated from ct values for mtDNA (ct_mtDNA_), and nuclear DNA (ct_nDNA_). The mitochondrial DNA content per cell was expressed as mtDNA copies per genome according to the formula 2 ^Δct^.

**Table 4:** Primer sequences for mtDNA copy number assessment.

Primer	Sequence (5’ → 3’)
**COX1_for**	CGGAGGAGGAGACCCCATTC
**COX1_rev**	TGGTAGCGGAGGTGAAATATGC
**ND3_for**	AGCCGCCGCCTGATACTG
**ND3_rev**	GGGGATATAGGGTCGAAGCCG
**POLG_for**	TCCTGTGGTCATTTATGGCA
**POLG_rev**	TAGATCCTGCCCACCCAAG
**RRM2B_for**	GCGATAATGCTGATGTCCAG
**RRM2B_rev**	CATAACCAAGCCGTAAGCAA

### Immunofluorescent (IF) staining

Cells were seeded onto glass coverslips and allowed to attach for 48 hours. Frozen tissue sections (6 μm) or cells on slides or coverslips were washed with PBS and fixed with 4% paraformaldehyde for 10 min. Subsequently samples were washed with PBS and permeabilized with PBS supplemented with 1% bovine serum albumin (BSA) and 0.2% Triton X-100 for 5 min. After a 30-min blocking step with PBS containing 1% BSA, samples were incubated with the antibody MTC02, which recognizes a mitochondrial protein of 60 kDa (dilution 1:200 for cells, 1:100 for tissue; Abcam, Cambridge, UK) and chicken anti cytokeratin 8/18 (dilution 1:300; Sigma) for 1 hour at 37°C. One sample of cells or tissue was incubated with mouse IgG1 isotype control (Dako) instead of primary antibody at the same concentration. After washing, coverslips were incubated with goat anti-mouse and goat anti-chicken fluorescently-labeled secondary antibodies (1:500; Life technologies, Carlsbad, CA). Samples were finally washed and mounted with Vectashield Hard Set mounting medium containing DAPI (Vector Laboratories, Burlingame, CA). Cells and mitochondria were visualized using fluorescent microscopy on a Zeiss Axio Imager M1. For quantification of MTCO2 staining, slides were scored automatically by using TissueQuest software (TissueGnostics, Vienna, Austria).

### Statistical analysis

All numerical data are presented as mean ±SEM from at least three independent experiments and were subjected to one-way ANOVA followed by Dunnett’s post-hoc test or Tukey's honest significant difference test (SPSS software version 20; SPSS Inc, Chicago, IL). Values were normalized to vehicle control (mock), which was set at 1.0. Statistically significant differences are denoted *, *P*<0.05; **, *P*<0.01; ***, *P*<0.001. *P*>0.05 was considered not significant (ns).

## Results

### Decreased glycolytic activity in cancer versus BE prostate epithelial cells

**Fig 1:**
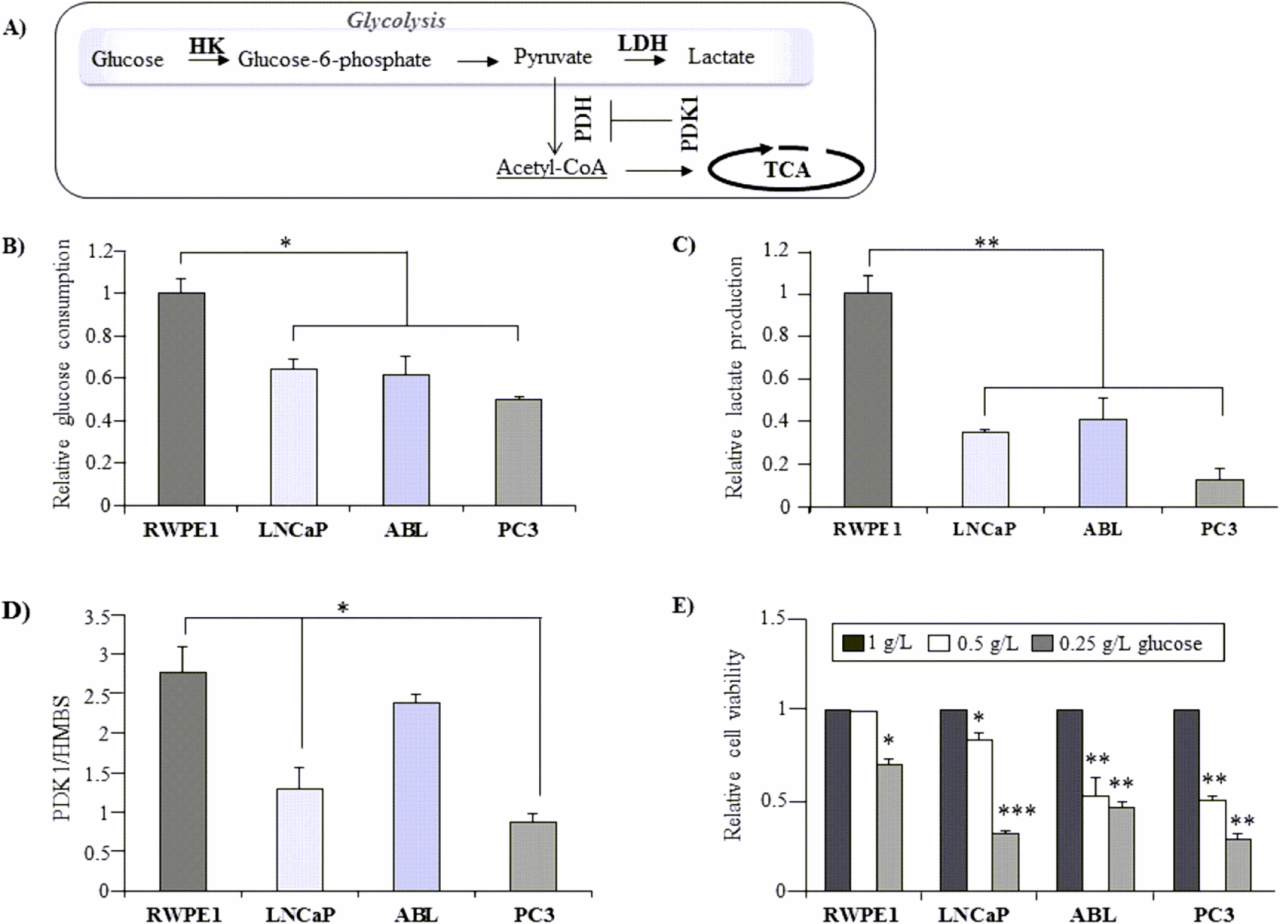
Differential glycolytic activity between BE and cancer cells of the prostate. (A) A simplified overview of cellular glucose metabolism. Glucose is metabolized to pyruvate, which is either converted to acetyl CoA via pyruvate dehydrogenase (PDH) for the TCA cycle or is metabolized to lactate by lactate dehydrogenase (LDH) and excreted. Pyruvate dehydrogenase kinase 1 (PDK1) is an enzyme, which inactivates the conversion of pyruvate to acetyl-CoA to fuel the TCA cycle thereby inhibiting glucose oxidation. To evaluate glucose consumption (B) and lactate production (C), BE prostate epithelial cells (RWPE-1) and PCa cell lines (LNCaP, ABL, and PC3) were seeded in triplicates in 6 well plates under standard culture conditions. Glucose and lactate levels were measured in the supernatant after 72 h. Values denote mean expression (±SEM) relative to levels in RWPE-1 cells (set as 1.0). (D) Basal mRNA expression levels of PDK1 were determined by means of qPCR. Values are denoted relative to the housekeeping gene hydroxyl-methyl-bilane synthase (HMBS). (E) To evaluate glucose dependence, cells were seeded in triplicates in 96 well plates with indicated concentrations of glucose for 72 h. Cell viability was assessed by WST-1 assay. Values were normalized to the vehicle control (mock) in normal growth media (1g/L glucose), which was set as 1.0. All results are presented as mean ±SEM from at least three independent experiments. Statistical significance is indicated (*, P < 0.05; **, P < 0.01; ***, P < 0.001).

Although it is widely accepted that altered glucose metabolism is associated with cancer development, the metabolic phenotype of cancer cells and in particular its regulation remain poorly understood. Therefore, we first investigated the glycolytic activity of BE and malignant prostate epithelial cell lines by measuring their glucose uptake and lactate production. A simplified overview of glucose metabolism is depicted in Fig 1A. When cells were maintained under standard glycolytic growth conditions (1 g/L glucose), BE prostate RWPE-1 cells consumed 1.71 ± 0.05-fold more glucose (*P* = 0.03) than LNCaP, ABL and PC3 PCa cell lines (Fig 1B). Under these conditions, RWPE-1 cells also produced significantly higher lactate levels (Fig 1C) (*P* = 0.008) compared to the PCa cell lines, indicating that BE RWPE-1 cells have a higher glycolytic activity than LNCaP, ABL and PC3 cells, respectively.

We next investigated basal expression levels of pyruvate dehydrogenase kinase (PDK1), a key regulatory protein of glycolysis that inhibits the uptake of pyruvate as acetyl-CoA into the TCA (Fig 1A). As shown in Fig 1D, expression of PDK1 was significantly increased by 3.15 fold ±0.11 (*P* = 0.03) and 2.13 fold ±0.20 (*P* = 0.02) in BE prostatic RWPE-1 cells compared to PC3 and LNCaP PCa cells, respectively. ABL cells also had lower PDK1 expression levels than RWPE-1 cells although the difference lacked statistical significance. This data again support the notion that benign RWPE-1 cells are more glycolytic than the PCa cell lines.

Despite the high glycolytic phenotype of RWPE-1 cells under standard growth conditions, their cell viability was interestingly less dependent on glucose than that of the PCa cell lines when cultured in glucose-restricted media (0.5–0.25 g/L glucose) (Fig 1E). Collectively, these results suggest that BE prostate cells apparently use glucose mainly for glycolysis and not for OXPHOS, as indicated by their low glucose dependence and high expression of PDK1.

### Dietary fatty acids increase cell viability of benign prostate RWPE-1 cells but not that of prostate cancer cell lines

**Fig 2:**
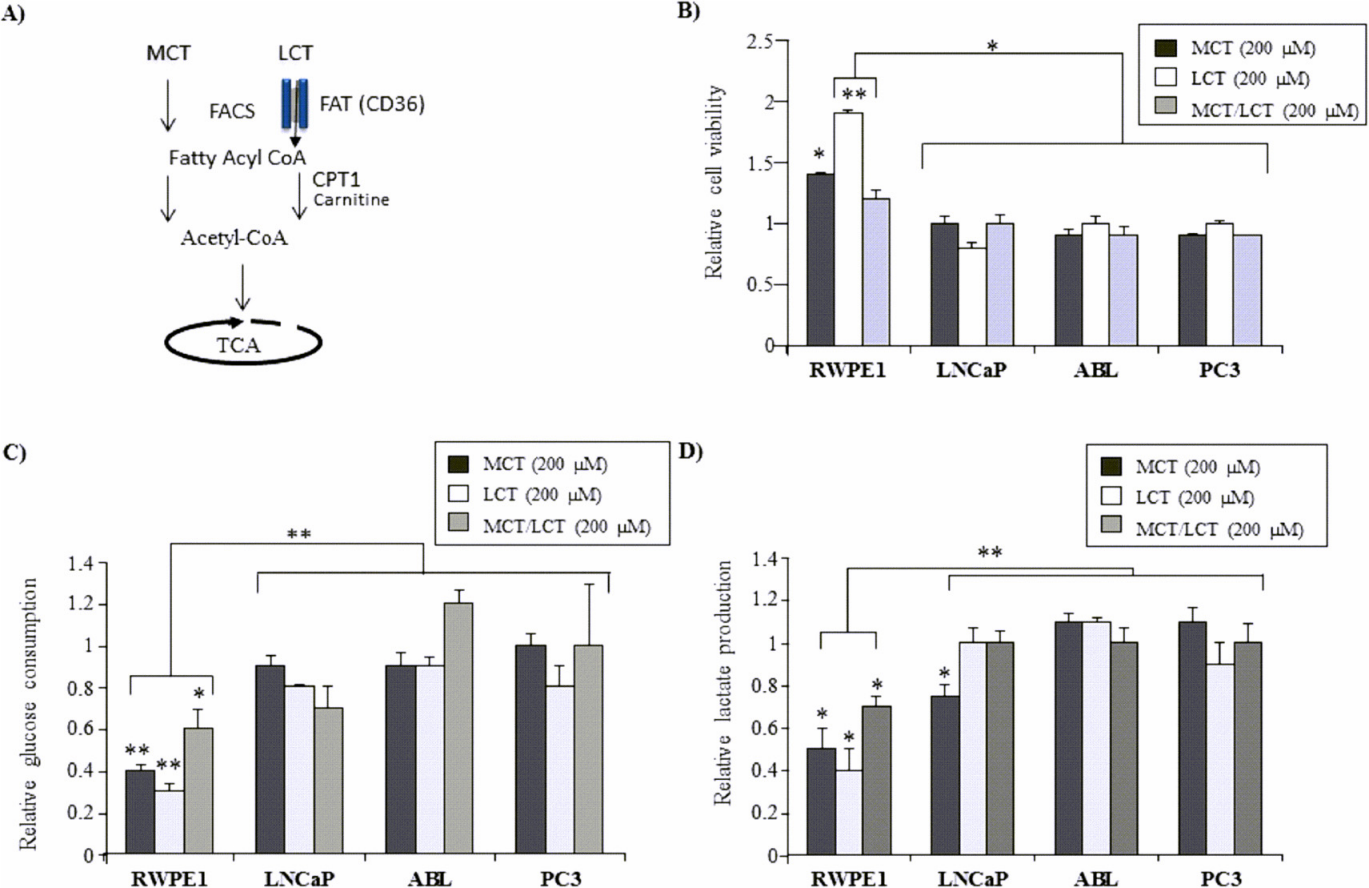
BE prostate epithelial cells utilize fatty acids as preferential energy source. (A) Simplified diagram of fatty acid (FA) catabolism. FAs such as MCTs and LCTs enter the cell via FA protein transporter such as CD36 (FAT, fatty acid translocase) or via direct diffusion, respectively. In the cell, they are converted to fatty acyl CoA by fatty acyl-CoA synthetase (FACS) and further transported into the mitochondria, a step which requires carnitine palmitoyltransferase 1 (CPT1) for the transport of LCTs across the inner mitochondrial membrane. Within the mitochondria, FAs are metabolized through the FA β-oxidation pathway resulting in the production of acetyl-CoA for the TCA cycle. (B) Effects of various FAs (MCTs, LCTs, and MCTs/LCTs) on the viability of BE (RWPE-1) and PCa (LNCaP, ABL, PC3) cells were evaluated by WST-1 assay. Cells were seeded in 96 well plates in triplicates and treated with 200 μM of the indicated FAs or vehicle (mock) for 72 h. Supernatants from the same experiment were subjected to assess (C) glucose consumption and (D) lactate production via enzymatic assays as described in material and methods. All values are normalized to vehicle control (mock), which was set at 1.0. Results are expressed as mean values (±SEM) from three independent experiments. Statistical significance is indicated (*, P < 0.05; **, P < 0.01).

In view of the aforementioned differences in glycolytic activities and the preference for lipid metabolism described in the literature, we next investigated the ability of BE and malignant prostate epithelial cells to use dietary fatty acids as energy source. We therefore applied MCT oils (MCTs, C-10) and LCT oils (LCTs, C-22) either alone or in combination (Table 1), respectively. In general, both, MCTs and LCTs, are catabolized to acetyl CoA mainly in the mitochondria, however, they differ in the way how they are transported into the cell and further on into the mitochondria (Fig 2A). As shown in Fig 2B, MCTs and LCTs increased cell viability of BE RWPE-1 cells over mock control with the strongest effect seen with 200 μM LCT within 72 hours. In particular, addition of 200 μM LCTs increased RWPE-1 cell viability by 1.95-fold ±0.01 (*P* = 0.008), MCT by 1.48-fold ±0.02 (*P* = 0.05) and the combination of MCT/LCT by 1.26-fold ±0.01 (*P* = 0.06), respectively, compared to the untreated mock control (Fig 2B). Notably, the strongest effect was observed with LCTs alone. By contrast, no growth-modulatory effect was observed on any of the tested PCa cell lines (LNCaP, ABL, PC3, and DuCaP) (Fig 2B and S2B Fig). Importantly, cell viability in BE RWPE-1 was enhanced already after a treatment period of 24 hours when no effect was detectable in PCa cells (S2A Fig). This is why the experimental treatment period was prolonged to 72 hours and was kept consistent for all further experiments.

In addition to increasing cell viability, MCTs and LCTs significantly decreased glucose consumption (MCT: 1.75-fold ±0.03, *P* = 0.007; LCT: 2.78-fold ±0.01, *P* = 0.005; MCT/LCT: 1.61-fold ±0.12, *P* = 0.04) and lactate production (MCT: -1.48-fold ±0.09, *P* = 0.02; LCT: -2.50-fold ±0.25, *P* = 0.01; MCT/LCT: -1.43-fold ±0.07, *P* = 0.05) in RWPE-1 cells relative to mock control (Fig 2C and 2D). Again, this effect was not observed in the PCa cell lines. There was only a moderate decrease in glucose consumption and lactate production in LNCaP cells upon treatment with MCTs (Fig 2C and 2D). However, this decrease was significantly less pronounced than the effect observed in RWPE-1 cells.

**Fig 3:**
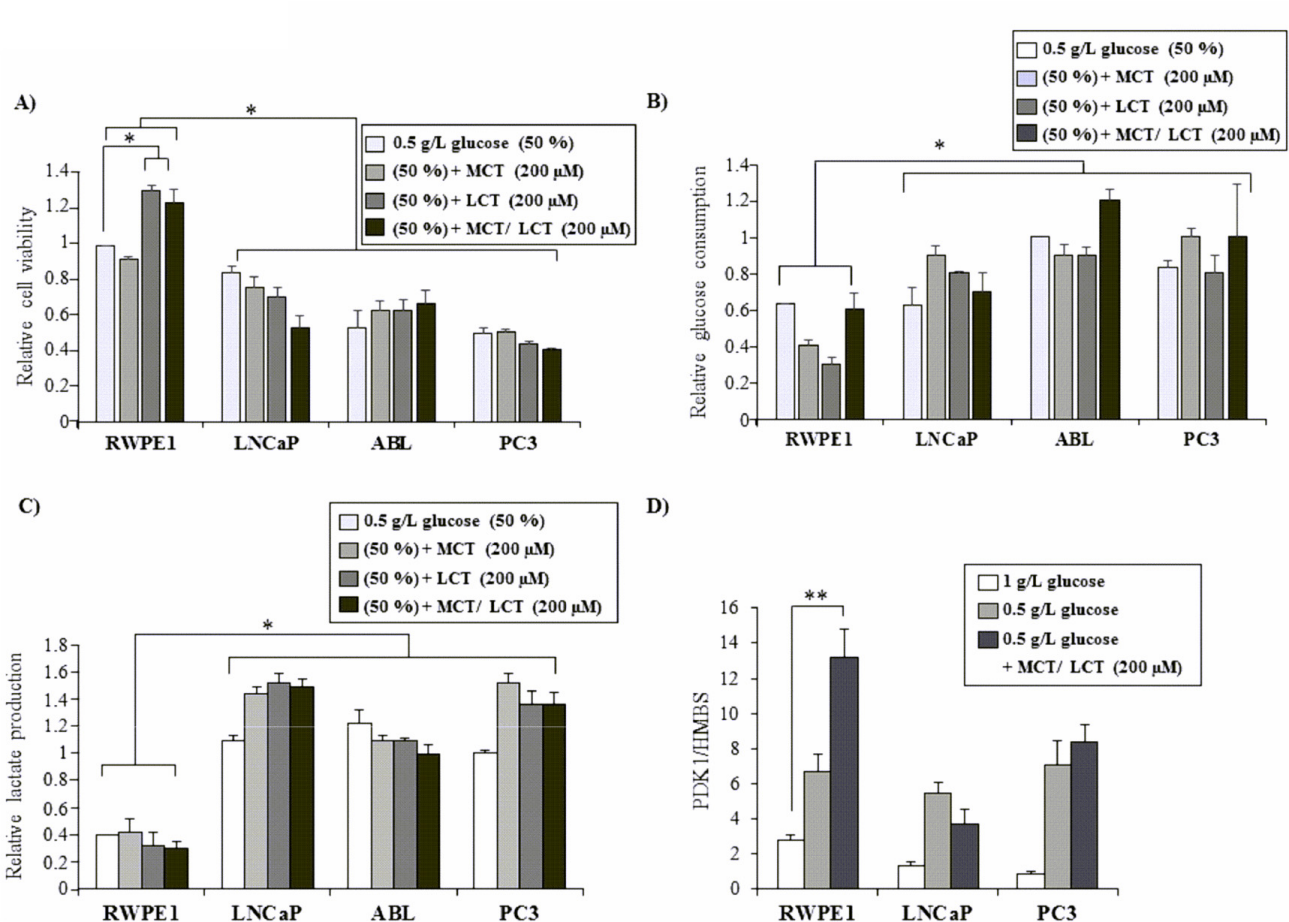
Effects of fatty acids under glucose-restricted culture conditions. BE RWPE-1 and PCa cells (LNCaP, ABL, and PC3) were grown in triplicates in 96 well plates for 24 h before being subjected to medium with reduced glucose levels (0.5 g/L) with or without 200 μM of MCTs, LCTs, MCTs/LCTs or equivalent vehicle (mock). (A) Cell viability was assessed by WST1 assay after 72 h. Cell culture supernatants of the same experiments were used to determine (B) glucose consumption and (C) lactate production using enzymatic assays as described in materials and methods. All values are normalized to vehicle control (mock) with standard growth conditions (1g/L glucose), which was set at 1.0. (D) PDK1 mRNA expression was determined by qPCR. Values denote mean expression (±SEM) relative to the housekeeping gene HMBS from three independent experiments. Statistical significance is indicated (*, P < 0.05; **, P < 0.01).

Because of the observed growth-stimulatory effect of MCTs and LCTs in RWPE-1 cells under standard culture conditions, we next examined whether cells used MCTs or LCTs as an alternative energy source when deprived of glucose. As shown in Fig 3A, cell viability of RWPE-1 cells also increased by 2.11-fold ±0.03 with LCTs (*P* = 0.02) and 1.98-fold ±0.07 with MCT/LCT (*P* = 0.04) (Fig 3A) when the glucose concentration in the medium was reduced to 0.5 g/L. However, no effect on cell viability was seen upon addition of MCTs alone, indicating that the growth stimulatory effect with MCT/LCT was likely caused by the LCTs. On the other hand, cell viability of the PCa cell lines, which were found to be more dependent on glucose as shown before (Fig 1E), was not stimulated by LCTs or MCTs, respectively, suggesting that they were not able to use MCTs or LCTs as energy source to compensate for glucose starvation.

Corresponding with this data, there was a significant decrease in glucose consumption (Fig 3B) and lactate production (Fig 3C) in RWPE-1 cells compared to the PCa cell lines (PC3, LNCaP, and ABL). Glucose starvation induced a strong increase in PDK1 expression in all cell lines (Fig 3D), demonstrating an activation of the glycolytic pathway under reduced glucose conditions. Of notice, PDK1 expression levels were further increased by MCT/LCT under glucose-reduced conditions in RWPE-1 cells by 1.97-fold ±0.09 (P = 0.005), an effect not seen in the PCa cell lines.

### Benign prostate epithelial RWPE-1 utilize the ketone body 3-OHB as an alternative energy source

**Fig 4:**
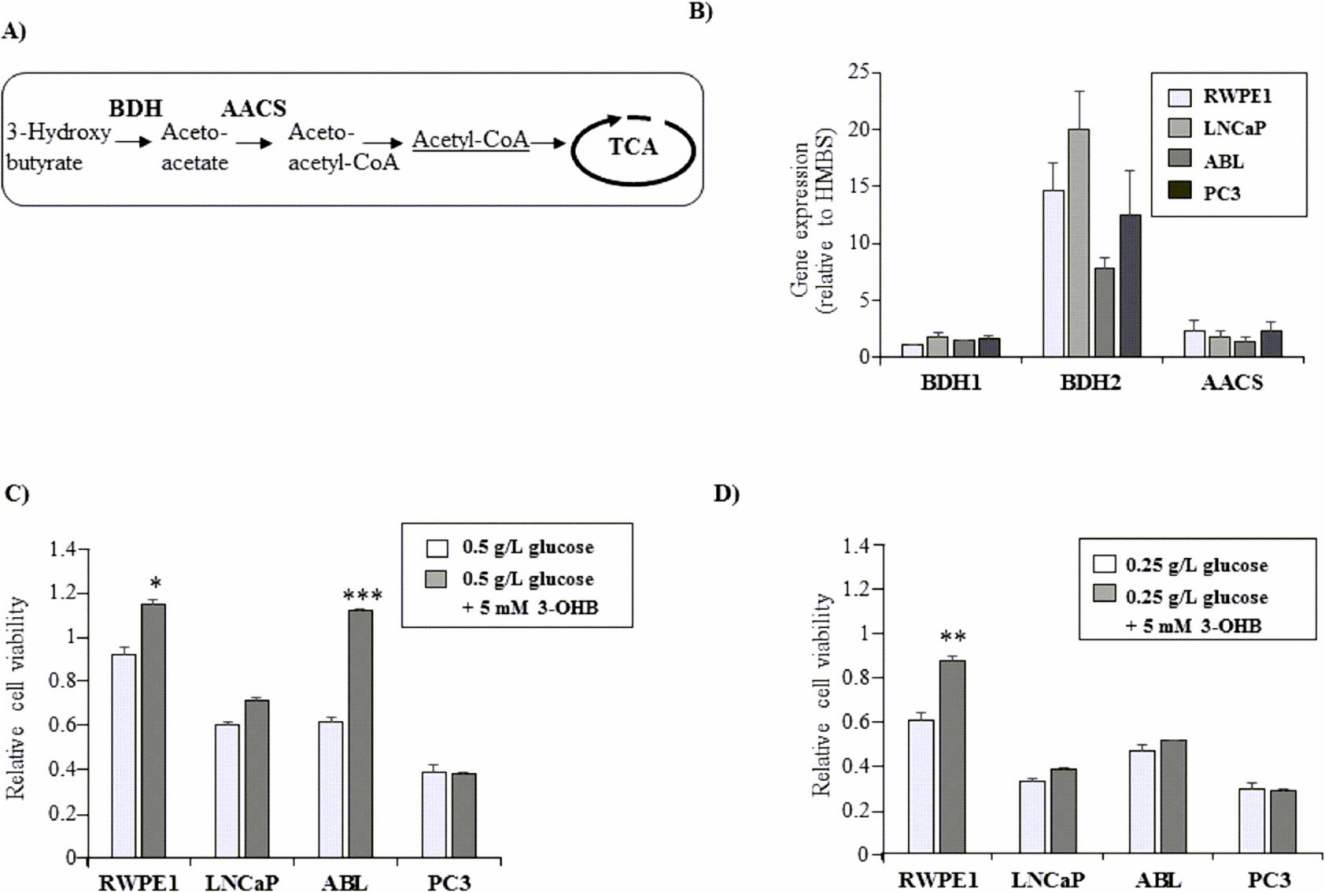
Effects of the ketone body 3-hydroxy butyrate (3-OHB) under glucose starvation. (A) Overview of cellular ketone body metabolism. Upon starvation, FAs are metabolized to ketone bodies in the liver, representing an important compensatory energy source for the cells. Oxidoreductase 3-hydroxybutyrate dehydrogenase (BDH) mediates the first step of ketone body degradation from 3-hydroxybutyrate into aceto-acetate, which is subsequently converted into two molecules of acetyl-CoA for TCA cycle. (B) Expression of BDH1, BDH2, and AACS was determined in BE (RWPE-1) and PCa cells (LNCaP, ABL, PC3) by qPCR and depicted as mean expression values relative to the housekeeping gene HMBS. (C and D) Effects of 3-OHB on cell viability under glucose starvation. Cells were cultured in 6-well plates in triplicates for 24 h prior to reduction of glucose concentrations to (C) 0.5 g/L or (D) 0.25 g/L in the absence (mock) or presence of 5 mM 3-OHB. Cell viability was assessed after 72 h using WST-1 assay. Values were normalized to vehicle control (mock) under standard growth conditions (1g/L glucose), which were set at 1.0. All results are expressed as mean values (±SEM) of three independent experiments. Significance is indicated (*, P < 0,05; **, P < 0.01; ***, P < 0.001).

In addition to directly acting on prostate cells, MCTs and LCTs may also act indirectly via the catabolism to ketone bodies [37]. Similar to the oxidation of MCTs and LCTs, ketone body catabolism culminates in the production of acetyl-CoA, which can enter the TCA cycle generating energy through the electron transfer system (Fig 4A). We therefore investigated the effects of 3-hydroxybutyrate (3-OHB), which is the most abundant ketone body produced by the liver upon glucose restriction. As shown in Fig 4B, the key enzymes of ketone body metabolism 3-hydroxybutyrate dehydrogenase type 1 and 2 (BDH1 and BDH2) and acetoacetyl-CoA synthetase (AACS) were found to be expressed in all prostate epithelial cell lines with BDH2 being the most abundant enzyme (Fig 4B), indicating that all tested prostatic cell lines are likely capable of catabolizing ketone bodies.

We next investigated the effects of 3-OHB (5 mM) on cell viability under glucose-reduced culture conditions (0.5 g/L and 0.25 g/L glucose). 3-OHB increased the cell viability of BE RWPE-1 cells by 1.25-fold ±0.11 (*P* = 0.005, Fig 3D) when grown with 0.5 g/L glucose and by 1.45-fold ±0.02 (*P* = 0.009, Fig 4D) when grown with 0.25 g/L glucose, suggesting that RWPE-1 cells are able to utilize ketone bodies as an energy source. Among the PCa cell lines, only ABL cells were rescued from glucose starvation-induced growth inhibition by 3-OHB. However, this effect disappeared when glucose concentrations were further reduced to 0.25 g/L (Fig 4C and 4D).

### Differential mitochondrial respiration in benign vs malignant cells of the prostate

The data thus far indicate that the metabolic demands of BE and malignant prostate epithelial cells are fundamentally different, with RWPE-1 cells not only being more glycolytic but also showing a higher preference for dietary MCT and LCT oils as a potential energy source, respectively. Given the marked differences between benign and cancer cells, high-resolution respirometry (HRR) was conducted to investigate the mitochondrial respiratory system that drives aerobic synthesis of ATP in the different cell lines under standard glucose conditions.

**Fig 5:**
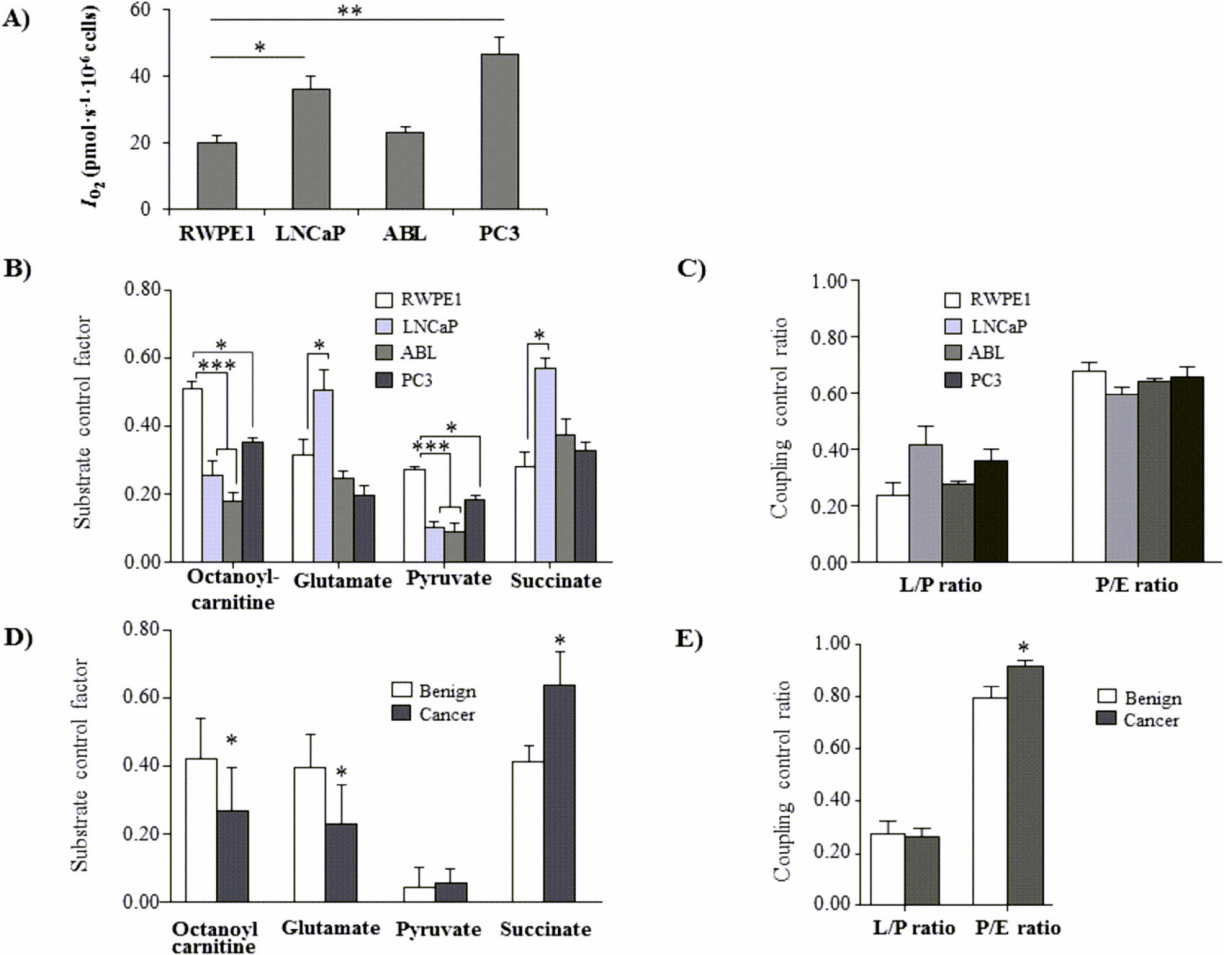
Differential OXPHOS capacities of BE and PCa cells. BE RWPE-1 and PCa cells (LNCaP, ABL, PC3) were cultured under standard culture conditions (1g/L glucose) for 48 h. (A) Base-line respiration (ROUTINE state) was measured in intact cells and expressed as O2 flow per cells, IO2 (pmol.s-1.10–6 cells). (B and D) Substrate control factor was assessed in permeabilized cells (B) or tissue (D) and indicates the relative increase of respiration measured after subsequent titration of octanoyl-carnitine, glutamate, pyruvate and succinate in the ADP-stimulated (OXPHOS) state. (C and E) Coupling control ratios were assessed in permeabilized cells (C) and tissues (E), respectively. The L/P ratio provides a degree for coupling efficiency and the P/E ratio embodies the relative limitation of OXPHOS capacity exerted by the phosphorylation system. All results are expressed as mean value (±SEM) of three independent experiments for cultured cell lines (n = 3) and six paired BE and cancer tissue samples (n = 6). Significance is indicated (*, P < 0.05; **, P < 0.01; ***, P < 0.001)

By measuring oxygen consumption in intact, non-permeabilized cells under standard glucose conditions, we found that the basal oxidative mitochondrial respiration (baseline respiration or ROUTINE state) was higher in the PCa cell lines LNCaP and PC3 compared to BE RWPE-1 cells (Fig 5A) whereas ABL PCa cells exhibited a base-line respiration similar to that of BE RWPE-1 cells. We next evaluated the respiratory capacity of pathways through the individual complexes of the electron transfer system (ETS) (with Complexes I-IV), which are located at the inner mitochondrial membrane [38]. To this end, cells were permeabilized with digitonin, followed by addition of malate and octanoyl carnitine (fatty acid ß-oxidation (FAO)-linked LEAK state in the absence of phosphorylation of ADP, FAO_*L*_). Addition of ADP at a saturating concentration enabled assessment of OXPHOS capacity (FAO_*P*_). Titration of glutamate, followed by pyruvate was performed to fuel electrons via Complex 1 (CI) into the Q-pool, thus allowing measurement of CI&FAO-linked respiration (CI&FAO_*P*_). Next, succinate was added for reconstitution of TCA cycle operation by feeding electrons simultaneously via CI&FAO and complex 2 into the Q-junction (CI&CII& FAO_*P*_). To evaluate the potential limitation of OXPHOS capacity by the phosphorylation system, a stepwise titration of the uncoupler CCCP was performed, allowing calculation of the P/E coupling control ratio [30].

As shown in Fig 5B, RWPE-1 cells show a significantly higher fatty acid oxidation (FAO) capacity after the addition of octanoylcarnitine than the three tested tumor cell lines, supporting our previous findings that BE RWPE-1 cells have a higher capacity to use fatty acids as energy source than PCa cells. Of notice, addition of glutamate had a significantly high impact on LNCaP PCa cells (glutamate control factor, Fig 5B), pointing out glutamine as a potential energy substrate. Interestingly, oxidation capacity after addition of glutamate was lower in ABL and PC3 cells. Furthermore, addition of pyruvate induced the highest increase in oxidative capacity in RWPE-1 cells (pyruvate control factor, Fig 5B), correlating with the high glycolytic capacity in the benign prostate cell line. The control of respiration exerted by CII (succinate control factor, Fig 5B) was significantly elevated in LNCaP compared to RWPE-1 cells but—similar to the effect seen with glutamate—declined in ABL and PC3 cells, which are not dependent on androgens compared to LNCaP cells. Although OXPHOS capacity is strongly limited by the phosphorylation system (expressed by low *P/E* ratios), no significant differences were observed among the different cell lines (Fig 5C).

Due to the limited number of appropriate cell lines mimicking BE prostate epithelial cells, we further wanted to confirm our *in vitro* findings in human prostate tissue. To this end, we performed high-resolution respirometry (HRR) on fresh prostate tissue biopsies obtained directly after radical prostatectomy, by applying the same titration regime used for the cell line-based experiments. For this purpose, paired benign and cancer samples were analyzed in parallel.

As summarized in Fig 5D, ß-oxidation of fatty acids following addition of octanoyl-carnitine leads to a significantly higher increase in respiration in benign prostate tissue when compared with cancer tissue, thus confirming our in vitro results. Addition of glutamate resulted in a lower oxidation capacity in cancer versus BE tissue, which was similar to the effect seen in ABL and PC3 cells. The addition of pyruvate did not cause an increase in respiration in either BE or cancer tissue (Fig 5D). Furthermore, the effect of succinate on respiration was significantly higher in cancer compared to BE tissue, resembling the respiratory phenotype observed in LNCaP cancer cells. The limitation of the OXPHOS capacity exerted by the phosphorylation system is significantly lower in cancer tissue, indicating a decrease in the apparent ETS excess capacity (Fig 5E).

### Differential mitochondrial content and mitochondrial genome copy numbers of benign vs malignant prostate epithelial cells of prostate

**Fig 6:**
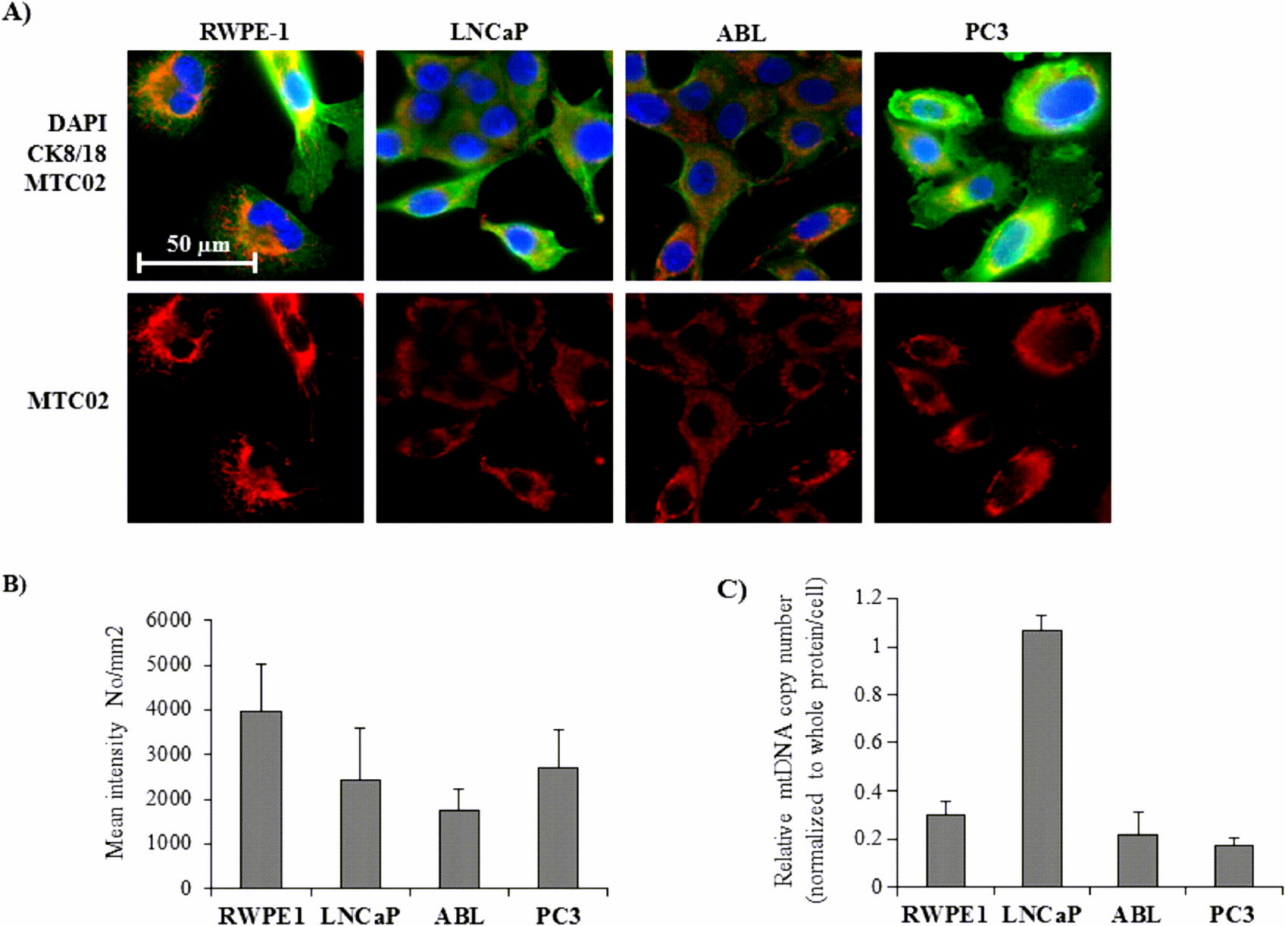
Mitochondrial mass and mtDNA contents in BE and malignant cells of the prostate. Mitochondrial mass in BE (RWPE-1) and malignant cells of the prostate (LNCaP, ABL, PC3) was determined by immunofluorescent staining of the 60 kDa mitochondrial glycosylated protein MTC02. (A) Representative pictures show the mitochondrial glycoprotein MTC02 in red. Counterstaining of nuclei (blue) and cytokeratin 8/18 (CK 8/18; green) were performed to determine cellular localization of mitochondria. Original magnification, x400. (B) MTC02 staining was quantified using TissueGnostics software. Values denote mean fluorescence intensity per mm^2^ (±SEM) of three independent experiments (n = 3). (C) Relative mtDNA copy number (COX1 and ND3 DNA fragments) was determined in relation to nuclear DNA (nDNA; POLG and RRM2B DNA fragments) in BE RWPE-1 and PCa cells (LNCaP, ABL, PC3) by qPCR. Results are denoted as mtDNA copy number following normalization to cellular protein content (Bradford) per cell to compensate for differences in cell size. Values are normalized to the cell line with the highest mtDNA copy number (LNCaP), which was set at 1.0. All results are expressed as mean value (±SEM) of three independent experiments.

Oxidative energy metabolism strongly depends on the quantity and quality of mitochondria, the cellular key organelles for energy production. Therefore, we next evaluated the mitochondrial content of BE and malignant prostate cell lines by immunofluorescent (IF) staining of the glycosylated mitochondrial protein MTC02. As shown in Fig 6A and 6B, we detected a lower staining intensity of MTC02 in PCa cell lines (LNCaP, ABL, PC3) compared with BE RWPE-1 cells with a non-significant decline with loss of hormone dependence in LNCaP and ABL (Fig 6A and 6B). In addition to MTC02 staining, we determined the mtDNA content per cell by means of qPCR by calculating the ratio of nuclear to mitochondrial DNA. mtDNA content was found to be highest in LNCaP cells, consistent with high oxygen consumption per cell (Fig 5A). Similar with the data obtained with MTC02 staining, the mtDNA content per cell appeared to decrease with the hormone dependence status of the cell lines (Fig 6C).

**Fig 7:**
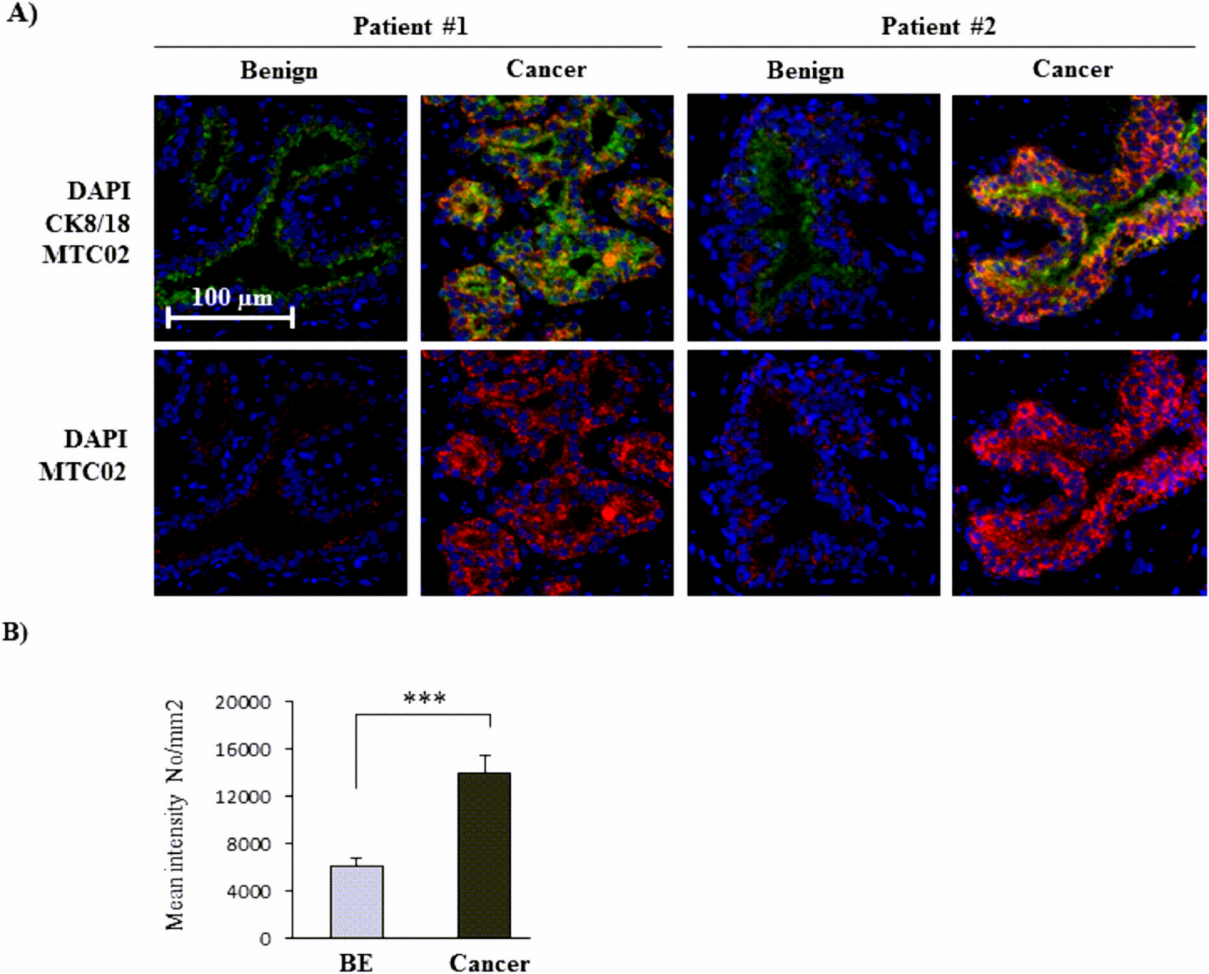
Mitochondrial mass in BE and malignant human prostate tissue. Immunofluorescent staining for MTC02 in human prostate tissue specimens. (A) Representative pictures show the mitochondrial glycoprotein MTC02 in red and cytokeratin 8/18 (CK 8/18) in green with DAPI counterstaining of nuclei (blue). Original magnification, x400. (B) MTC02 staining was quantified using TissueGnostics software. Values denote mean fluorescence intensity per mm^2^ (±SEM) of 8 BE and 13 cancer areas from 2 different patients. Significance is indicated (***, *P* < 0.001).

In human prostate tissue, on the other hand, we found a significantly mean fluorescence staining intensity for MTC02 in cancer (14013.16 ± 1417.96) compared to benign tissue (6087.01 ± 765.01, *P* = 0.001) (Fig 7A and 7B).

## Discussion

This study aimed to investigate the differences of the metabolic phenotype between BE and malignant cells of the prostate with a specific focus on the bioenergetics use of dietary MCT and LCT oils. To this end, we evaluated cellular glycolytic activity and mitochondrial oxidative respiration when offering glucose or various FAs as energy sources. We report that BE prostate epithelial cells (RWPE-1) prefer to utilize dietary FAs as energy source compared to PCa cells (LNCaP, ABL, PC3). Moreover, RWPE-1 was the only cell line relieved from glucose starvation-restricted growth by the addition of the ketone body 3-OHB, which is a major product of FA catabolism in the liver, culminating in the production of acetyl-CoA, which then enters the TCA cycle [37] (Fig 4A). This stimulating effect of FAs on cell viability of BE prostate cells was further supported by OXPHOS measurements demonstrating that RWPE-1 cells indeed show a significantly higher ß-oxidation of FAs than PCa cells although, overall, the tumor cells had a 2-fold higher base-line respiration activity than BE cells. We were able to confirm this finding in vivo by showing that benign prostate tissue has a significantly higher ß-oxidation activity than cancer tissue, similar to the results obtained with the cell lines.

Many types of cancers have an enhanced glycolytic activity compared to BE cells, a metabolic phenotype that enables highly proliferating cells to cope for their increased energy demands (reviewed in [39,40]). In contrast, PCa cells more likely have a lipid-driven phenotype, which is characterized by a preferential utilization of energy substrates for ß-oxidation [41].

In this study, we show that under standard culture conditions, BE RWPE-1 prostate epithelial cells have a higher glycolytic activity than PCa cells (LNCaP, ABL, and PC3) as demonstrated by higher levels of relative glucose consumption and lactate production, respectively. One possible reason might be the inhibition of m-aconitase by zinc, resulting in an accumulation of citrate and reduced TCA cycle activity. Indeed, zinc accumulation decreases upon malignant transformation [42] and secreted citrate levels are significantly higher in prostatic fluid from normal than from malignant prostates [43,44].

The high glycolytic activity of BE RWPE-1 cells in our study was further supported by higher expression levels of PDK1 compared with PCa cells. This enzyme inhibits pyruvate dehydrogenase (PDH), which is the rate limiting enzyme for glucose oxidation by regulating the direction of pyruvate into the TCA cycle. Of notice, the inhibition of PDH by PDK1 was previously reported to correlate with increased FA oxidation [45]. In addition, we found that BE RWPE-1 cells–although more glycolytic—were less sensitive to glucose starvation than PCa cells, a finding also reported by others [46].

Liu and coworkers have shown previously that prostate epithelial cells preferentially utilize FAs over glucose as bioenergetics source [11]. In particular, their study showed that prostate cells show an increased cellular uptake of ^3^H-palmitic acid over ^3^H-glucose with no significant differences between BE (RWPE-1) and cancer cells (LNCaP, PC3). In this study, we observed that the viability of BE prostate RWPE-1 cells is significantly increased by MCTs or LCTs, an effect not seen in the PCa cell lines tested. Moreover, RWPE-1 but not the tumor cells were able to use especially LCTs to compensate for glucose starvation-induced growth inhibition. Upon malignant transformation of prostate cells, it seems that cells switch their metabolism to burn citrate via m-aconitase, which is fed into the TCA cycle to increase OXPHOS. Mitochondrial respiratory measurements undertaken in our study support this notion, since in fact, the PCa cell lines tested show an increased base-line respiration compared to BE RWPE-1 cells. Hence, our data indicates that under standard culture conditions, PCa cells use OXPHOS and the TCA cycle for energy production from glucose. Of notice, only ABL cells had a base-line respiration similar to BE RWPE-1 cells. However, this may be due to maintenance of ABL cells in an androgen-deprived medium and the resulting slower growth rate compared to parental LNCaP.

OXPHOS measurements further supported our notion that BE RWPE-1 cells have a high glycolytic phenotype and a preferential use of FAs as energy source than PCa cells, since their octanoyl-carnitine fed oxidation capacity was significantly higher. Importantly, we were able to confirm our *in vitro* findings in human prostate tissue. HRR on tissue specimens mirrored the cell line results with significantly higher octanoyl-carnitine induced respiration in BE prostate compared to PCa. Furthermore, respiration in PCa tissue compared to BE prostate tissue was significantly higher after addition of succinate, resembling the respiratory phenotype of LNCaP cells. In the androgen-independent cell lines, which represent more advanced and metastatic stages of PCa, this effect seemed to decline. Pyruvate-driven oxidation was higher in BE RWPE-1 than in PCa cell lines. In tissue, pyruvate-driven oxidation was low with only a marginal difference between BE and cancer tissue. Similar results have been reported by others, demonstrating that glucose, rather than lipid-derived acetyl-CoA, is the predominant substrate for energy production of CA cells under cell culture conditions [47,48]. In summary, our results suggest that PCa cells undergo metabolic changes that decrease the utilization of FAs and increase the dependence on glucose, a phenomenon that is most pronounced in castration-resistant ABL and androgen-independent PC3 cells.

Increased glutaminolysis counts as another intriguing hallmark of cancer, providing proliferating tumor cells with energy and precursors for various different anabolic pathways, including *de novo* lipid synthesis (reviewed in [49]). A recently published study by Dasgupta and colleagues [50] has reported on glutamine as an essential substrate for *de novo* lipid synthesis in PCa cells. Our HRR measurements showed a significant increase in glutamate-driven respiration in LNCaP cells compared to BE RWPE-1 cells. By contrast, glutamate-driven respiration was lower in ABL and PC3 cells than in RWPE-1 and was also lower in PCa tissue compared to BE prostate. Succinate-driven respiration on the other hand was significantly higher in PCa tissue compared to BE prostate, which is in accordance with a significant increase in respiratory activity in response to succinate in PCa tissue and a marginal increase in ABL and PC3 compared to BE RWPE-1 cells.

OXPHOS capacity depends on the quantity and quality of mitochondria. Notably, alterations in mtDNA content have been suggested to play an important role in PCa [51–53]. In particular, several studies reported on reduced amounts of mtDNA in the androgen-independent cell lines C4-2, PC3, and DU145 compared to parental LNCaP cells, suggesting that reduction of mtDNA shifts PCa cells to androgen dependence and epithelial to mesenchymal transition changes, which then may lead to tumor progression [51,54,55]. Consistent with this data, we detected significantly fewer mtDNA copies in ABL versus LNCaP cells. In addition, AR negative PC3 and RWPE-1 cells had lower amounts of mtDNA than AR positive LNCaP. In a recent study Grupp and coworkers investigated the impact of mitochondrial content by staining for MTC02 in PCa tissue samples and found a correlation of MTC02 with ERG positive PCa with increased staining intensity in PTEN negative tumor samples within the ERG positive subset [56]. We therefore also investigated the impact of mitochondrial content by immunofluorescent staining of MTC02 in the different prostate cell lines as well as in human prostate tissue samples. In human PCa tissue, mitochondrial mass was significantly higher in cancer than in benign tissue, confirming the findings of Grupp et al. In the cell lines used in our study, however, MTC02 staining was even lower in the PCa cell lines LNCaP, ABL and PC3 compared with benign RPWE1 cells and did not correlate with mtDNA data. However, base-line respiratory activity correlated with mitochondrial amount among the three PCa cell lines with highest levels in PC3 cells in both measurements. Only RWPE-1 cells had a higher mitochondrial mass but a lower base-line respiratory activity than PCa cells. In that respect, it must be taken into account that the levels of basal oxidative mitochondrial respiration is largely dependent on cell size, mitochondrial density and expression levels of OXPHOS proteins, which are likely different among the cell lines. Moreover, it is known that morphology, number, and function of mitochondria is regulated by various different cellular events such as autophagy, fusion and fission, and mitochondrial proteolysis [57], which may all hamper the interpretation of our data. In a previous study, Higgins et al reported on marked differences in glycolytic and OXPHOS activities among LNCaP, DU145 and PC3 cells [58]. A limitation of using immortalized benign cell lines such as RWPE-1 is also their low expression of AR and their androgen-independent but proliferative basal/intermediate phenotype as recently reviewed in [59], which may probably result in discrepant results between cell lines and human tissue. Hence, overall, it should be considered that the metabolic phenotype strongly varies among the different cell lines and does not always reflect the situation of PCa *in vivo*, a fact that should be considered when conducting metabolic studies in cell lines.

Collectively, data herein suggest that the preference for dietary FAs in BE cells of the prostate may be exploited for a diet concept, where cancer cells are starved while BE cells are provided with dietary FAs. Observational studies have been undertaken in the past, which have investigated associations between dietary FAs and the risk of PCa [17,60]. A meta-analysis—although not providing highly significant data—revealed that alpha-linoleic acid (ALA) seems to reduce the risk of PCa whereas polyunsaturated FAs do not have any significant effect. Furthermore, it should be considered that dietary FA like MCTs and LCTs lead to the production of high amounts of ketone bodies in the liver [18,37]. Hence, MCTs and LCTs may not directly but indirectly act on prostate cells through the production of ketone bodies. Several studies have reported that a ketogenic diet, based on glucose starvation and the resulting high levels of ketone bodies, is associated with a positive effect on several types of cancers [61–64]. In our study, the ketone body 3–OHB, in a concentration range measured in serum upon a ketogenic diet (reviewed in [65]), exerted similar effects on proliferation and cell viability as those observed with MCTs and LCTs.

In summary, this study shows that PCa cells have a lower glycolytic activity than BE prostate cells characterized by a decreased glucose consumption and lactate production, but active mitochondria with a high base-line respiration fueled by glucose. In addition, BE prostate cells have a high preference to utilize dietary FAs as energy source, a phenomenon that we also confirmed for human prostate tissue. Further studies are warranted to determine whether these bioenergetics differences can be exploited for the development of new diagnostic or therapeutic anti-cancer strategies.

## Supporting Information

S1 Fig Histology of prostate tissue samples. HE staining showing biopsy cores that were taken for OXPHOS measurements from benign prostate (BE) and prostate cancer (CA). Representative images were taken from samples of one patient. Biopsy cores are indicated by arrows (magnification 25x). P63/P504S staining was performed to differentiate between benign and malignant prostate (p63 brown, P504S red) as shown by an overview of the whole slide (C) (magnification 25x) and at a higher magnification (200x) of the framed area (D).
(PPTX)

S2 Fig Effects of FAs on cell viability. (A) Different FAs (MCTs, LCTs, and MCTs/LCTs) were added to BE (RWPE-1) and PCa (LNCaP, ABL, PC3) cells at a final concentration of 200 μM and cell viability was evaluated by WST-1 assay 24 h afterwards. (B) DuCaP cells were incubated with 200 μM of MCTs, LCTs, and MCTs/LCTs for 72 h. Cell viability was evaluated by WST-1 assay. All values were normalized to vehicle control (mock), which was set at 1.0. Results are expressed as mean values (±SEM).
(PPTX)
