# Supplementary figures and images for: Differential Utilization of Dietary Fatty Acids in Benign and Malignant Cells of the Prostate
Source: PLoS One. 2015 Aug 18;10(8):e0135704. doi: 10.1371/journal.pone.0135704 (PMC4540467; doi:10.1371/journal.pone.0135704)

## Slide 1
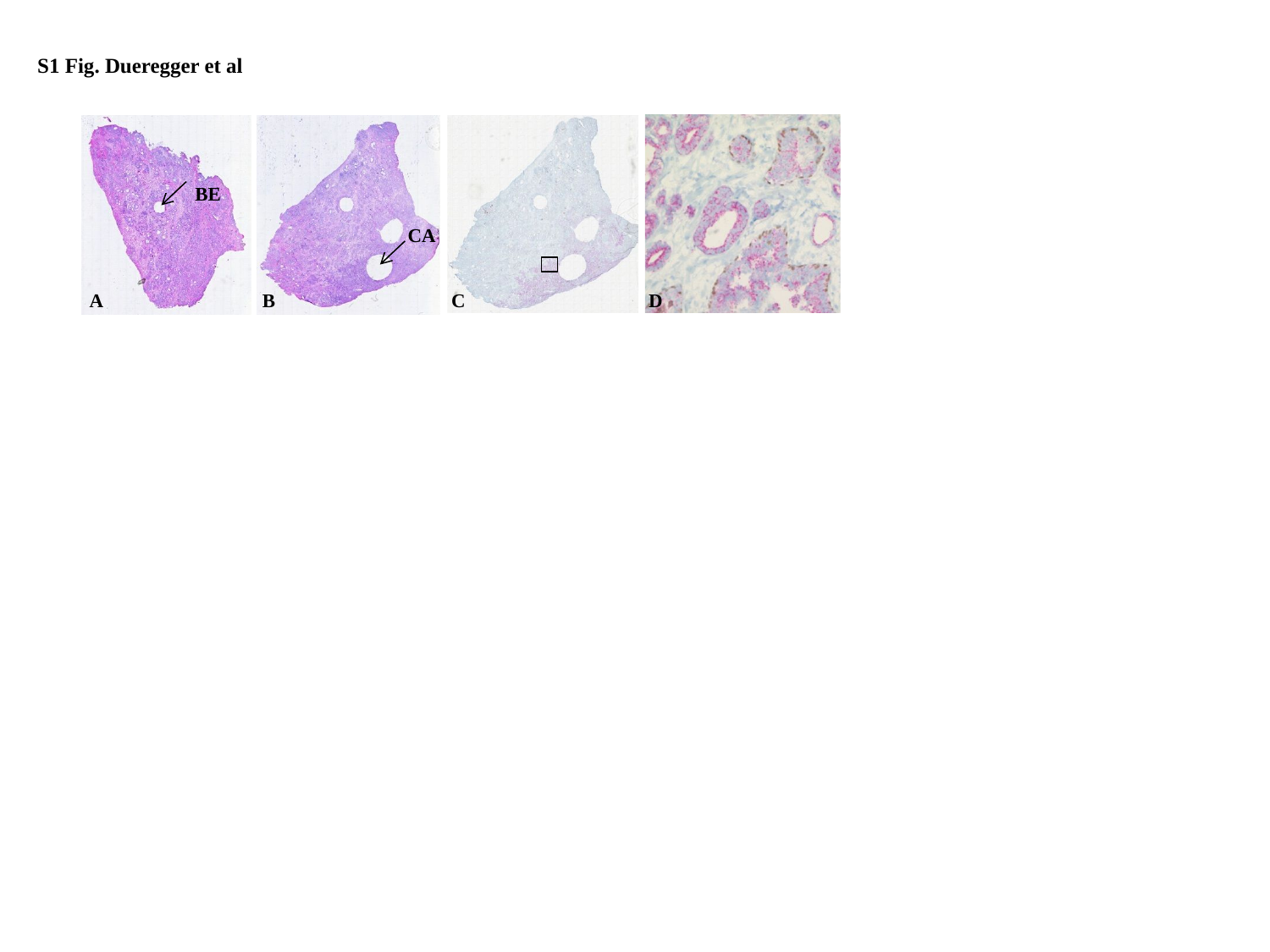

S1 Fig. Dueregger et al
BE
CA
B
C
A
D

Supplement: S1 Fig — HE staining showing biopsy cores that were taken for OXPHOS measurements from benign prostate (BE) and prostate cancer (CA). Representative images were taken from samples of one patient. Biopsy cores are indicated by arrows (magnification 25x). P63/P504S staining was performed to differentiate between benign and malignant prostate (p63 brown, P504S red) as shown by an overview of the whole slide (C) (magnification 25x) and at a higher magnification (200x) of the framed area (D). (PPTX) [file pone.0135704.s001.pptx]
