# Supplementary material for: Differential Utilization of Dietary Fatty Acids in Benign and Malignant Cells of the Prostate
Source: PLoS One. 2015 Aug 18;10(8):e0135704. doi: 10.1371/journal.pone.0135704 (PMC4540467; doi:10.1371/journal.pone.0135704)
Supplement: S2 Fig — (A) Different FAs (MCTs, LCTs, and MCTs/LCTs) were added to BE (RWPE-1) and PCa (LNCaP, ABL, PC3) cells at a final concentration of 200 μM and cell viability was evaluated by WST-1 assay 24 h afterwards. (B) DuCaP cells were incubated with 200 μM of MCTs, LCTs, and MCTs/LCTs for 72 h. Cell viability was evaluated by WST-1 assay. All values were normalized to vehicle control (mock), which was set at 1.0. Results are expressed as mean values (±SEM). (PPTX) [file pone.0135704.s002.pptx]

## Slide 1
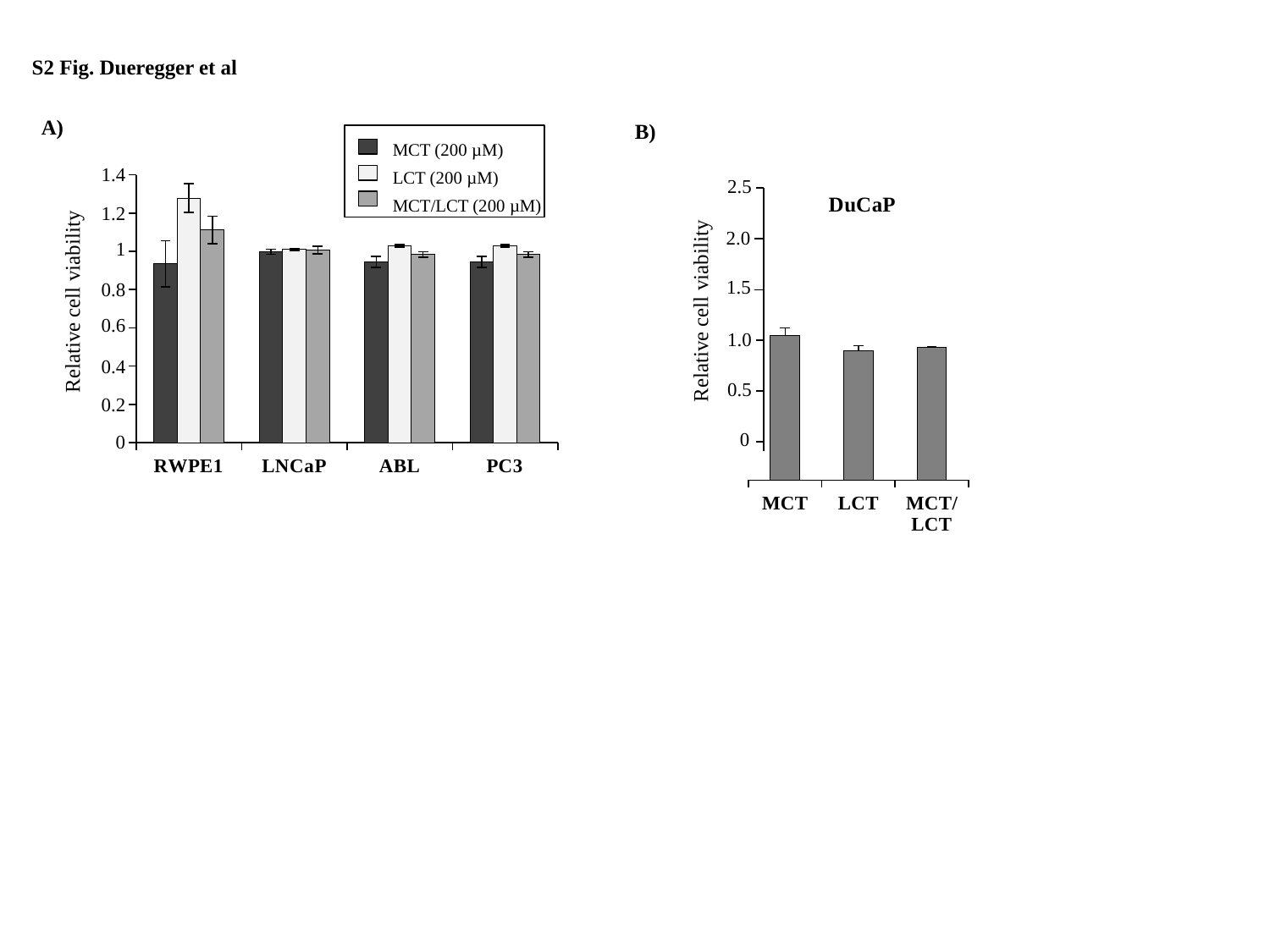

S2 Fig. Dueregger et al
### Chart:
| Category | DuCaP |
|---|---|
| MCT | 1.1279391424619643 |
| LCT | 1.0096818810511756 |
| MCT/LCT | 1.033195020746888 |A)
### Chart
| Category | | | |
|---|---|---|---|
| RWPE1 | 0.9350503919372899 | 1.2777155655095185 | 1.110862262038074 |
| LNCaP | 0.9972183588317106 | 1.008692628650904 | 1.0073018080667593 |
| ABL | 0.943930991990142 | 1.0286506469500927 | 0.9836722119531733 |
| PC3 | 0.943930991990142 | 1.0286506469500927 | 0.9836722119531733 |
MCT (200 µM)
LCT (200 µM)
MCT/LCT (200 µM)
1.4
1.2
1
0.8
Relative cell viability
0.6
0.4
0.2
0
B)
2.5
2.0
1.5
Relative cell viability
1.0
0.5
0
